# Changes in Gait Parameters and Gait Variability in Young Adults during a Cognitive Task While Slope and Flat Walking

**DOI:** 10.3390/healthcare8010030

**Published:** 2020-02-03

**Authors:** Ga Young Park, Sang Seok Yeo, Young Chan Kwon, Hyeong Seok Song, Yu Jin Lim, Yu Mi Ha, Seung Hee Han, Seunghue Oh

**Affiliations:** 1 Department of Physical Therapy, Graduate School, Dankook University, Dandae-ro, Dongnam-gu, Cheonan-si, Chungnam 330-714, Korea; pgy0614@hanmail.net; 2 Department of Physical Therapy, College of Health Sciences, Dankook University, 119, Dandae-ro, Dongnam-gu, Cheonan-si, Chungnam 330-714, Korea; eangbul@hanmail.net (S.S.Y.); dydcksgud3@naver.com (Y.C.K.); aszx7474@naver.com (H.S.S.); dladbwls6887@naver.com (Y.J.L.); dbal721@gmail.com (Y.M.H.); gkstmdgml200@naver.com (S.H.H.)

**Keywords:** slope gait, dual-tasking, spatiotemporal gait parameters, gait variability

## Abstract

This study investigates the effects of a cognitive task while walking on a slope or a flat surface on gait parameters and gait variability in young adults. The participants consisted of thirty healthy young subjects. They were instructed to walk on a slope or on a flat surface while performing or not performing a cognitive task, which involved speaking a four-syllable word in reverse. A wearable inertia measurement unit (IMU) system was used to measure spatiotemporal parameters and gait variability. Flat gait (FG) while performing the cognitive task (FGC) and uphill gait (UG) while performing the cognitive task (UGC) significantly altered stride times, gait speeds, and cadence as compared with FG and UG, respectively. Downhill gait (DG) while performing the cognitive task (DGC) caused no significant difference as compared with DG. Gait variability comparisons showed no significant difference between UGC and UG or between FGC and FG, respectively. On the other hand, variabilities of stride times and gait speeds were significantly greater for DGC than DG. FGC and UGC induce natural changes in spatiotemporal gait parameters that enable the cognitive task to be performed safely. DGC should be regarded as high complexity tasks involving greater gait variability to reduce fall risk.

## 1. Introduction

Gait refers to movements of the body from one point to another through systematic and repetitive alternating movements of the limbs and trunk [1,2,3]. Gait requires body stability and balance and is maintained by a complex process controlled by the central nervous system [4,5]. It is generally thought that the repetitive alternating movements of gait do not require a high level of attention [2,6]. However, walking during difficult conditions such as downhill or on icy roads requires considerable attention to maintain balance and minimize fall risk [5,7]. Cognitive dual-tasking requires the simultaneous execution of two tasks and requires cognitive factors [8,9]. Cognitive tasks have been reported to affect movement when they require movement and attention [10,11], and cognitive tasks that require much attention during gait influence gait patterns [6,12]. Gait velocity has been reported to decrease and gait variability to increase while performing a cognitive task like counting backwards [13]. Gait variability is an inherent natural fluctuation that occurs during continuous gait cycles and is a predictor of fall risk [14,15,16]. Therefore, reductions in cognitive function reduce postural control and attention and cause gait pattern changes that increase fall risk [17,18].

Previous studies on the relationship between gait and cognitive function have usually been conducted on a flat floor [6,19,20]. However, in daily life gait, different environments are encountered, such as slopes, that can be more slippery than flat floor, which require higher levels of balance [21,22,23]. Also, it has been demonstrated that center of mass (COM) is changed to maintain balance during gait on a slope [24,25], which means more biomechanical control is required during gait on a slope than on a flat surface [26,27]. Previous studies have shown that step length and cadence increase during uphill gait (UG) in healthy adults, and conversely, that step length and cadence decrease during downhill gait (DG) [11,28]. In particular, decreased velocity, step length, and stride length during DG are associated with fear of falling and have different patterns from those on flat ground due to the increased fall risk. In fact, slope gait has been reported to be an environmental factor that increases fall risk [29,30]. Although slope gait while performing a cognitive task is an important consideration when analyzing gait patterns and can indicate the need for gait training to reduce the risks of falls, few studies have investigated the effect of a challenging cognitive task on slope gait. Especially, there is no study regarding DGC in slope walking for spatiotemporal parameters and gait variability presenting fall risk.

The purpose of this study is to investigate the effects of slope gait while performing a cognitive task on spatiotemporal parameters and gait variability in young adults.

## 2. Materials and Methods

### 2.1. Subjects

Thirty healthy subjects without any musculoskeletal or nervous system disease participated in the present study. Participant demographics are summarized in Table 1. Healthy adults were chosen to observe changes in spatiotemporal gait parameters during slope walking while performing a cognitive task. The following inclusion criteria were adopted: (1) the absence of any lesion that might have affected the experiment; (2) the ability to understand and follow the experiment; and (3) no history of lower limb or spinal orthopedic surgery. The study was explained in detail to all participants before commencement and all agreed to participate in the experiment. The experiment was conducted with the Institutional Review Board (IRB, 2019-11-015-002) approval of Dankook University.

### 2.2. Apparatus

To evaluate spatiotemporal parameters and gait variability, five wearable inertia measurement unit (IMU) systems (LEGSys+, BioSensics, Cambridge, MA, USA) were used. The spatiotemporal parameters investigated included stride time, stride length, stride speed, and gait variability, which included stride time, stride length, and stride speed variability. Sensors were attached using a strap at the midpoint of both posterior iliac spine, 3 cm above both knees, and 3 cm above both ankles [31]. The sensor sampling rate was 100 Hz.

The slope walkway used was 2.75 m long, 0.91 m wide, and 0.53 m height at either end. The walkway had a steel frame surface and was raised using a gantry and held in position by clamps at an angle of 11 degrees. Previous study reported that critical inclination at which walking speed changed was found between 9 and 12 degrees [28]. Thus, we choose the 11 degrees. Note that walking upwards is denoted by a positive angle. Flat gait (FG) was performed on flat surface of the same length and width as the walkway.

### 2.3. Protocol

**Figure 1:**
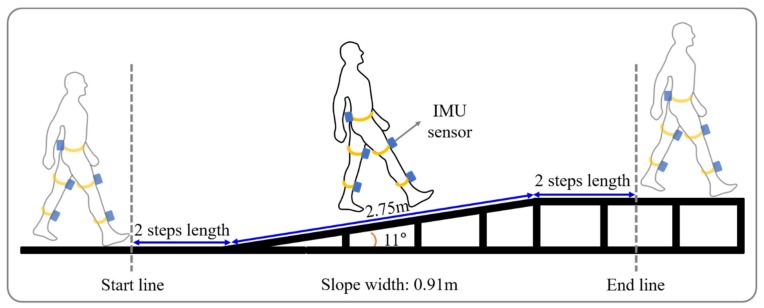
Schematic diagram of ramp walking.

Subjects conducted six tasks, namely, uphill gait (UG), uphill gait while performing a cognitive task (UGC), downhill gait (DG), downhill gait while performing a cognitive task (DGC), FG, and while performing a cognitive task (FGC). Each task was performed 3 times. Briefly, with a subject standing on a line 2 steps before the ramp, we gave the verbal instruction “start”. Subjects were also asked to take 2 more steps after clearing the ramp (Figure 1). If a subject made a mistake or gave up a task, the task was repeated. For each task, data was acquired regarding a minimum of two strides.

### 2.4. The Cognitive Task

The cognitive task involved speaking a word with four syllables in reverse (e.g., A.S.I.S → S.I.S.A). The reason we have selected the cognitive task, which is spelling a word with four syllables in reverse, was to use the working memories of subjects. When humans use their working memories, the premotor cortex gets activated [32]. When humans perform the gait, the premotor cortex also gets activated [33]. Hence, we select the cognitive task [34] to provide workload to the same brain area.

The word was provided verbally when a subject started a task. Subjects were instructed to provide an answer before clearing the ramp or the flat walkway. If a subject answered after the ramp or flat walkway, he/she was asked to repeat the task.

### 2.5. Data Analysis

Spatiotemporal gait parameters were averages of those measured during the three, repeat trials. Data obtained before and after the ends of tasks were excluded. Note that the ramp used in our study consists of a flat surface before and after the ramp (Figure 1); data obtained before and after the ends of tasks were excluded. The Shapiro-Wilk test was performed for the evaluation of the distribution of the variables. The Mann-Whitney *U*-test determined the significances of differences between with and without performing the cognitive task as follows: in uphill gait stride time, cadence and variability of gait speed, in downhill gait stance phase and variability of stride length, in flat gait variability of stride time. The independent *t*-test was used to determine the significances of difference in spatiotemporal parameters and gait variability based on the assumption of normality. The independent *t*-test was used to determine the significances of difference in spatiotemporal parameters and gait variability. The analysis was conducted SPSS Ver. 21.0 (SPSS Inc., Chicago, IL, USA), and statistical significance was accepted for *p*-values < 0.05. 

## 3. Results

**Figure 2:**
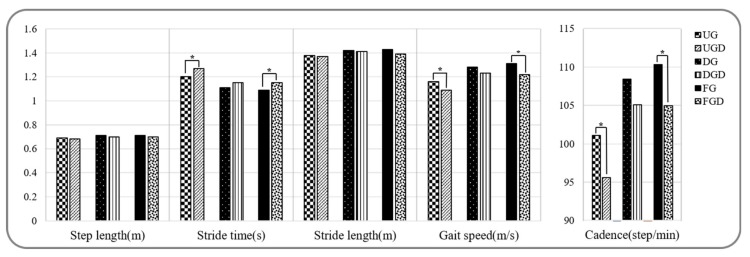
Comparison of spatiotemporal gait parameters while performing or not the cognitive task. UG: uphill gait, UGD: uphill gait with dual-task, DG: downhill gait, DGD: downhill gait with dual-task, FG: flat gait, FGD: flat gait with dual-task. * *p* < 0.05.

**Figure 3:**
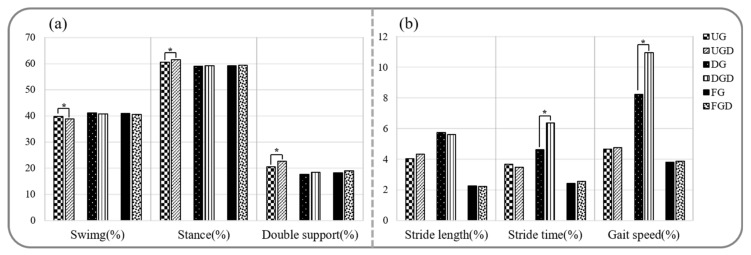
Comparison of spatiotemporal gait parameters and gait variabilities while performing or not the cognitive task. (**a**) Comparison of spatiotemporal gait parameters. (**b**) Comparison of gait variabilities. UG: uphill gait, UGD: uphill gait with dual-task, DG: downhill gait, DGD: downhill gait with dual-task, FG: flat gait, FGD: flat gait with dual-task. * *p* < 0.05.

Spatiotemporal parameters during walking and walking with a cognitive dual-task are provided in Figure 2 and Figure 3a. Stride time, stance phase, and double support phase while UGC were significantly greater than UG, but gait speed, cadence, and the swing phase were significantly decreased (*p* < 0.05). In contrast, no significant difference was observed between spatiotemporal parameters for DGC and DG (*p* > 0.05). Stride times while FGC were significantly greater and gait speed and cadence were significantly lower than while FG (*p* < 0.05).

The comparison of gait variabilities while walking with or without performing the cognitive task is shown in Figure 3b. Comparisons between UGC and UG or FGC and FG revealed no significant difference in gait variabilities of stride length, stride time, and gait speed (*p* > 0.05). In contrast, gait variabilities of stride time and gait speed were significantly greater for DGC than for DG (*p* < 0.05).

## 4. Discussion

In this study, we investigated changes in spatiotemporal gait parameters and gait variability during walking on a slope or a flat surface while performing or not performing a cognitive task in young adults. For spatiotemporal parameters, stride times were greater and gait speed and cadence were lower for the UGC and FGC tasks than for the UG and FG tasks. On the other hand, no significant difference was observed between spatiotemporal parameters for the DGC and DG tasks. However, variabilities in stride time and gait speed were significantly greater for the DGC task than the DG task. Consequently, our findings suggest that DGC increases gait variability, and thus, increases fall risk.

Previous studies that investigated the effects of performing a cognitive task while walking [5,17,18,35,36] have reported slower gait speeds [37,38,39], reduced cadence [39,40], shorter stride lengths [38,39], increased stride times [39] during FGC. In 2017, Soangra et al. reported that reductions in step length, gait speed, and cadence are strategic compensations aimed at optimizing gait pattern stability and minimizing additional attentional demands [11]. These results are consistent with our findings, which show dual-tasking reduces gait speed and stride length and increases stride time. Consequently, FGC induced natural changes in spatiotemporal gait parameters to enable dual-tasking to be performed safely.

Results of the UGC task were similar to FGC task results; that is, stride time increased, and gait speed and cadence decreased. In addition, UDC reduced the swing phase and increased the stance and double support phases. Although no previous study has examined the effect of UGC on spatiotemporal parameters, UG-related changes in spatiotemporal gait and dual task-related changes during FG have been reported previously in young and older healthy adults [25,38,39,41]. Previous studies have reported UG requires a different lower extremity motor pattern because the body tends to be pulled passively backwards by gravity [42,43]. Generally, spatiotemporal parameters changes, including decreased cadence [44] and gait speed [16,45] and increased stride length [22,45], are observed during UG. In 2013, Richard et al. demonstrated the different effects of FG and UG on spatiotemporal gait parameters among elderly adults and reported the double support phase was greater during UG than during FG [41]. In 2017, Soangra et al. reported that stance phase and double stance phase were increased during FGC [11]. With the exception of stride length, these findings are similar result to those obtained during the present study. In previous studies, stride length was increased during UG [22,45], and in the present study, stride length was smaller during UGC than during UG; reduced stride length would be closely related with optimizing the stability of the gait pattern [11,46]. Thus, UGC and FGC appeared to induce similar gait changes.

The DGC task showed increases in gait variability, including stride time and gait speed, as compared with the DG task, which is known to be inherently less stable than FG or UG tasks [25,27] because gravity tends to push the body forward. This means greater modulation of gait pattern is required to counteract this passive physical effect to reduce fall risk [43]. Generally, DG induced reductions in gait speed and step length and increased gait variability as compared with FG [16,24,28]. However, our result only showed increased variability in stride times and gait speeds during DGC as compared with DG. Several studies have reported that gait variability is closely related to fall risk [15,16,47,48]. In 1996, Sun et al. report that increased gait variability is the result of the modulations of stride time and speed to prevent falling [22]. On the other hand, another study reported a change in the variability of COM displacement during DGC [25]. Yahya et al. demonstrated COM displacement during DGC using a treadmill, and found decreased gait speed and increased variability of COM displacement during DGC as compared with DG [25]. COM displacement is closely related to lower limb movement [43], and thus, increased variability in COM displacement can be regarded as a manifestation of a changed gait pattern. This result suggests that DGC requires additional effort to reduce fall risk [22]. Consequently, DGC can be regarded as a task of high complexity requiring increased gait variability and gait pattern modulation.

The present study has a number of limitations that warrant consideration. First, only young adults were included, which limits the generalizability of our results, and these findings need to be evaluated on old adults and patients who need gait rehabilitation for direct use in a clinical environment. Second, we only analyzed two strides during gait due to the length of the ramp used, although we believe the ramp realistically reflected situations encountered in daily life.

## 5. Conclusions

The results of this study showed that performing cognitive tasks while walking affects spatiotemporal parameters and gait variability. In particular, dual-tasking was found to result in significant changes in spatiotemporal gait parameters but not gait variability during the FGC and UGC tasks as compared with the FG and UG. In contrast, the DGC task only showed an increase in gait variability as compared with the DG task. Therefore, it appears that the FGC and UGC tasks induced natural changes in spatiotemporal gait parameters to enable dual-tasking to be performed safely. On the other hand, DGC can be regarded as a task of high complexity that evokes a compensatory strategy to reduce fall risk. We believe our findings improve understanding of gait pattern changes induced by cognitive tasks while walking on slopes and flat surface, and clinicians who need to adjust the gait training complexity for their patients can consider the DGC training, which can provide attentive training, having high complexity to induce compensatory strategy in the aspect of task-specific training.

## Figures and Tables

**Table 1:** General characteristics of subjects.

Gender	Number of Subjects	Age	Height (cm)	Weight (kg)
Male	10	25.00 (1.94)	170.70 (7.18)	71.10 (11.99)
Female	20	22.15 (1.39)	162.10 (4.85)	52.80 (4.82)
Total	30	23.10 (2.07)	164.97 (6.96)	58.90 (11.70)

Values represent mean (±standard deviation).
